# Evaluation of Quality of Life and Satisfaction in Patients with Fixed Prostheses on Zygomatic Implants Compared with the All-on-Four Concept: A Prospective Randomized Clinical Study

**DOI:** 10.3390/ijerph18073426

**Published:** 2021-03-25

**Authors:** Juan Alberto Fernández-Ruiz, Mariano Sánchez-Siles, Yolanda Guerrero-Sánchez, Jesús Pato-Mourelo, Fabio Camacho-Alonso

**Affiliations:** 1 Clinica Fernández, 07800 Ibiza, Spain; administracion@clinicafernandez.es; 2 Clínica Dental Puerta Nueva, 30001 Murcia, Spain; marianosasi@yahoo.es; 3 Department of Anatomy and Psychobiology, University of Murcia, 30100 Murcia, Spain; 4 Galimplant, 27600 Sarria (Lugo), Spain; jpatomourelo@hotmail.com; 5 Department of Oral Surgery, University of Murcia, 30100 Murcia, Spain; fcamacho@um.es

**Keywords:** quality of life, satisfaction, fixed prostheses, zygomatic implants, all-on-four concept

## Abstract

*Purpose*: No published research has compared patients’ quality of life and satisfaction with fixed prostheses supported by zygomatic implants with those supported by all-on-four prostheses. The aim of this study was to evaluate patients’ quality of life and satisfaction with fixed prostheses on zygomatic implants compared with the all-on-four concept. *Materials and Methods*: A total of 80 patients with atrophic edentulous maxillae were randomized into two groups: Group 1 (rehabilitated with fixed prostheses supported by 2–4 zygomatic and 2–4 conventional implants in the anterior region) and Group 2 (fixed prostheses on four implants in the anterior region following an all-on-four concept). One year after placement of the definitive prostheses, patients completed OHIP-14 and satisfaction questionnaires. *Results*: In all seven domains of the OHIP-14 and in the overall scores, a worse quality of life was found in Group 2 patients, with statistically significant differences between the two groups (*p* ≤ 0.05). Patients with zygomatic implants were more satisfied with their prostheses, with a statistically significant difference (*p* < 0.001). *Conclusions*: According to the results of this study, rehabilitation of patients with edentulous atrophic maxillae with prostheses supported by zygomatic implants combined with anterior implants provided better patient quality of life and satisfaction than prostheses supported by four implants.

## 1. Introduction

In the field of implant dentistry, rehabilitation of severely atrophic maxillae derived secondary to loss of teeth has presented major challenges for clinicians and patients. Following the loss of molars, a process of advancing sinus pneumatization and expansion begins in the maxillary posterior region [1]. When placing posterior implants, the most widely used approach to this problem is maxillary sinus lifting combined with bone grafts and guided tissue regeneration to increase the vertical dimension of the bone available for implant insertion [2]. Procedures using autologous bone may be used, but this is particularly invasive as bone must be harvested from extraoral sites. Moreover, this approach will generate morbidity, risks, and complications such as loss of the graft, economic cost, technical difficulty, infection, and time needed for the graft to integrate [3].

As an alternative to these complex and invasive techniques, the use of anatomical buttresses and residual bone is a predictable way to rehabilitate the atrophic maxilla with dental implants [4,5]. This approach avoids the complications and morbidity associated with bone grafting, reduces treatment costs and time, and results in high patient overall satisfaction [6,7]. In this context, zygomatic implants were first introduced by Higuchi [8] and Brånemark et al. [9] to support prosthetic rehabilitations in patients who had suffered maxillary atrophy by tumors, malformation, or trauma.

The ultimate objective of these surgical techniques—bone regeneration or angled implant placement—is to restore patients’ masticatory function, esthetics, comfort, and to improve self-esteem and social ease [6]. Implant supported fixed prostheses meet all these objectives, leading to higher levels of patient satisfaction with treatment, as well as higher success rates [7].

The zygomatic fixture is the result of developments in reconstructive techniques for fixed prosthetic rehabilitation of patients with extensive maxillary defects using longer implants (≥30 mm). Zygomatic implants (one or two in each maxilla) are fixed in the body of the malar prominence. They are usually combined (except in cases of extreme maxillary atrophy) with 2–4 conventional implants in the maxillary bone of the anterior region [10,11]. According to the literature, immediate prosthetic loading in patients with zygomatic implants offers positive and predictable outcomes [12], with high survival rates in the long term (96.7–100%) [13,14,15,16,17,18,19,20]. However, post-surgical complications have been reported, the most frequent being maxillary sinusitis (1.5–14%), followed by soft-tissue infection (2%), paresthesia (1%), and oroantral fistulas (0.4%) [13,21,22,23].

Another alternative to bone grafting in the posterior region of atrophic maxillae is to place four implants in the maxillary anterior region, immediately loaded with provisional fixed prostheses [24]. Of these four implants, the two anterior implants are placed vertically, while the two posterior implants are placed at angles of between 35° and 45° to avoid the anterior wall of the maxillary sinus. Implant platforms are positioned as posteriorly as possible to minimize the prosthetic cantilevers; this has been described as the all-on-four concept [24,25,26,27,28]. Like zygomatic implants, immediate prosthetic loading of four implants in the all-on-four protocol has been successful. Definitive fixed full-arch prostheses are generally placed within 6–12 months [29].

For most people, poor oral health impacts on their general quality of life, and so this variable constitutes an important aspect of evaluating the outcome of this type of prosthetic rehabilitation [30]. The oral health impact profile (OHIP-14) [31,32,33] is a 14-item questionnaire (a shortened version of the OHIP-27) designed to evaluate the impact of oral health on quality of life. The index measures people’s perception of the social impact of oral disorders on their well-being. It investigates only negative impacts, whereas some other oral-health-dependent quality-of-life instruments capture both positive and negative impacts. However, the OHIP is the most frequently used instrument and remains the best documented [34]. In addition to the influence of prosthetic rehabilitations on patients’ quality of life, it is also important to determine their level of satisfaction/dissatisfaction [35,36].

Nevertheless, to date no prospective randomized clinical study has assessed the quality of life and satisfaction of patients rehabilitated with fixed prostheses on zygomatic implants compared with implants and fixed prostheses treated per the all-on-four concept.

The aim of this study was to evaluate quality of life and satisfaction in patients treated with fixed prostheses supported by one or two zygomatic implants combined with two to four anterior implants, compared with patients with fixed prostheses supported with four implants in the anterior region of the maxilla (all-on-four concept).

## 2. Materials and Methods

### 2.1. Recruitment and Patient Characteristics

The study protocol was approved by the University of Murcia Ethics Committee (2674/2019) and was carried out between July 2017 and January 2020 at two centers: the University Dental Clinic (University of Murcia, Murcia, Spain) and a private dental clinic. The research followed the requirements for the performance of clinical trials based on the Consolidated Standards of Reporting Trials (CONSORT statement). Subjects were treated according to guidelines established by the Declaration of Helsinki for medical research involving human subjects. All subjects provided their informed consent to participate.

Inclusion criteria were as follows: patients aged over 18 years; with complete edentulism in the maxilla or some residual teeth with indications for tooth extraction; diagnosed by cone-beam computed tomography (CBCT) with severe maxillary atrophy not suitable for rehabilitation with conventional implant supported prostheses; medically healthy subjects with absence of medical contraindications for oral surgical procedures (ASA I/II); patients willing to provide informed consent to take part in the study.

Exclusion criteria were as follows: presence of acute maxillary sinusitis; antecedents of trauma involving zygomatic bone fracture; presence of some disease, condition, or medication that could compromise healing or osteointegration (diabetes mellitus, bisphosphonate administration, or severe osteoporosis); presence of severe mental disorder; patients who had received radiotherapy of the head and neck during the previous 18 months; patients unwilling to provide informed consent to take part in the trial.

None of the patients who fulfilled the inclusion criteria and were invited to take part in this trial refused to do so. To calculate a representative sample size, a power of 80% was required (5% alpha level). A total of 80 patients with atrophic maxillary edentulism were included in this prospective randomized clinical study.

Regarding the mandibular situation, all the patients included in the study presented adequate conditions that would not affect the quality of life after the treatments carried out in the study. All of them were in stable conditions at the level of occlusion at the time of starting the study and the OHIP-14. The trial was not publicly registered due to the data protection rules in Spain.

### 2.2. Study Groups

All patients underwent clinical and radiological examinations with CBCT scans (Romexis Planmeca, Helsinki, Finland). All patients were diagnosed with severe maxillary atrophy; this made them unsuitable for rehabilitation by means of conventional implants supporting fixed prostheses. The patients were randomized into two groups (*n* = 40 per group) using the online service www.randomization.com.

Group 1: patients received 2–4 zygomatic implants and 2–4 anterior implants (Galimplant S.L., Sarria, Spain) with internal connection. Surgery was performed under general anesthesia with nasal intubation and local infiltration with lidocaine and epinephrine (1:50,000) blocking the posterior, nasopalatine, anterior palatine, and infraorbital nerves. Implants were placed with insertion torque values between 45 and 60 Ncm.

Group 2: patients received four conventional implants in the premaxilla (Galimplant S.L., Sarria, Spain) with internal connection. Of these four implants, two anterior implants were placed vertically; the other two were angled between 35° and 45° to avoid the anterior wall face of the maxillary sinus and so that the implant platforms were positioned as posteriorly as possible in order to reduce prosthetic cantilevering (all-on-four concept). Implants were placed with insertion torque values between 45 and 55 Ncm. Surgery was performed under IV sedation and local anesthesia with lidocaine and epinephrine (1:50,000) blocking the posterior, nasopalatine, anterior palatine, and infraorbital nerve nerves.

In both groups, after suturing mucoperiosteal flaps with non-absorbable 4/0 suture (Supramid, Aragó, Spain), impressions of both jaws and jaw relation registrations were carried out immediately after surgery to fabricate an immediate provisional fixed prosthesis in resin (theses prostheses were delivered during the 48 h after surgery). The patients were prescribed postoperative antibiotics and analgesics. Six months after surgery, the provisional prostheses were replaced with definitive metal–resin fixed prostheses (all prostheses were screw-retained). Oral hygiene instructions for at home were provided to all patients.

During the entire trial period, patients received regular check-ups to detect post-surgical complications (maxillary sinusitis, paresthesia, oroantral fistulas, or orbital cellulitis). Individual clinical and radiological analyses of all implants were performed using a digital radiography system (RVG model 5100, Kodak, Rochester, NY, USA) for early detection of any possible bone loss or peri-implantitis (changes in the level of the crestal bone conjunction with bleeding on probing, with or without concomitant deepening of peri-implant pockets, mobility, and presence of pus) [23]. The prostheses were also evaluated for any problems such as prosthetic fracture or accumulation of food trapped apical to the prostheses.

At least one year after definitive prosthesis placement, all patients completed the OHIP-14 and satisfaction questionnaires about their implant-supported fixed prostheses.

### 2.3. Oral Health Impact Profile-14

The OHIP questionnaire (in its short version) was applied to detect changes in oral quality of life. It consists of 14 items that explore different aspects of oral function and quality of life. Patients are questioned about problems relating to speaking, taste perception, eating discomfort, and problems with dentures. The total score ranges from 0 to 56, where higher scores correspond to poorer oral quality of life [32].

### 2.4. Level of Satisfaction with Prosthesis

To assess the degree of satisfaction with the prostheses, patients completed a simple questionnaire with five grades for the question “how satisfied are you with your implant-supported prostheses?”: dissatisfied, unchanged, slightly satisfied, satisfied, and extremely satisfied [34].

### 2.5. Statistical Analysis

Data were analyzed using the SPSS version 20.0 statistical package (SPSS^®^ Inc., Chicago, IL, USA). A descriptive study was made of each variable. The associations between the different qualitative variables were analyzed using Pearson’s chi-squared test. Student’s *t*-test for two independent samples was applied to quantitative variables, in each case determining whether variances were homogeneous. Statistical significance was established as *p* ≤ 0.05.

## 3. Results

**Figure 1:**
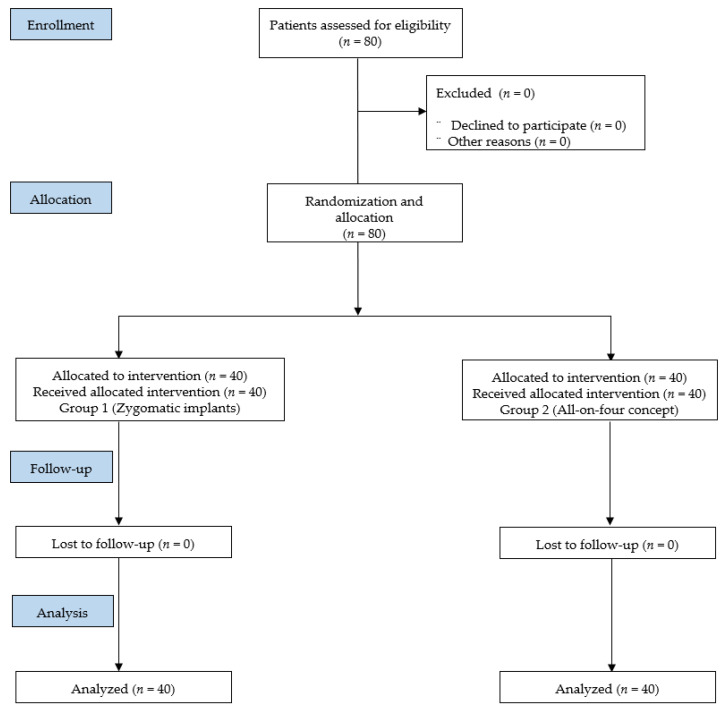
Flowchart of patient recruitment into this study according to the Consolidated Standards of Reporting Trials (CONSORT) statement.

This prospective randomized clinical trial recruited a total of 80 patients, as shown in the CONSORT flowchart in Figure 1. The follow-up time after definitive prosthetic placement was 19.40 ± 4.37 (12.00–22.00) months in Group 1 (zygomatic implants) and 20.25 ± 3.01 (12.00–24.00) months in Group 2 (all-on-four), without statistically significant differences between the groups (*p* = 0.314). The two groups were homogeneous in terms of demographic characteristics (age: *p* = 0.684; sex: *p* = 0.117); toxic habits (smoking: *p* = 0.749; alcohol consumption: *p* = 0.090), and the type of antagonist (*p* = 0.679), natural teeth being the most frequent in both groups (Table 1).

Regarding implant distribution, a total of 396 implants were placed, 236 in Group 1 (139 zygomatic implants and 97 conventional implants), and 160 in Group 2 (all-on-four). The details of implant lengths and diameters are shown in Table 2. According to the classification system developed by Aparicio et al. [13], of the 139 zygomatic implants placed, there were no cases of ZAGA (i.e., zygoma anatomy-guided approach protocol) 0, 3, or 4; 24 cases (17.27%) of ZAGA 1; and 115 cases (82.73%) of ZAGA 2.

No post-surgical complications occurred in patients treated with the all-on-four concept, while two patients treated with zygomatic implants presented complications: one (2.50%) case of maxillary sinusitis and one (2.50%) case of orbital cellulitis. Maxillary sinusitis was resolved by administering nonsurgical treatment and antibiotics, and orbital cellulitis was resolved spontaneously with time.

Regarding peri-implant variables, no cases of peri-implantitis appeared among the 97 conventional implants in Group 1. Of the 139 zygomatic implants placed in Group 1, 17 (12.23%) showed signs of peri-implantitis. In patients treated with the all-on-four concept, 19 of 160 implants presented peri-implantitis (11.87%). No significant differences in the incidence of peri-implantitis were found between the two groups (*p* = 0.113) (Table 3).

**Figure 2:**
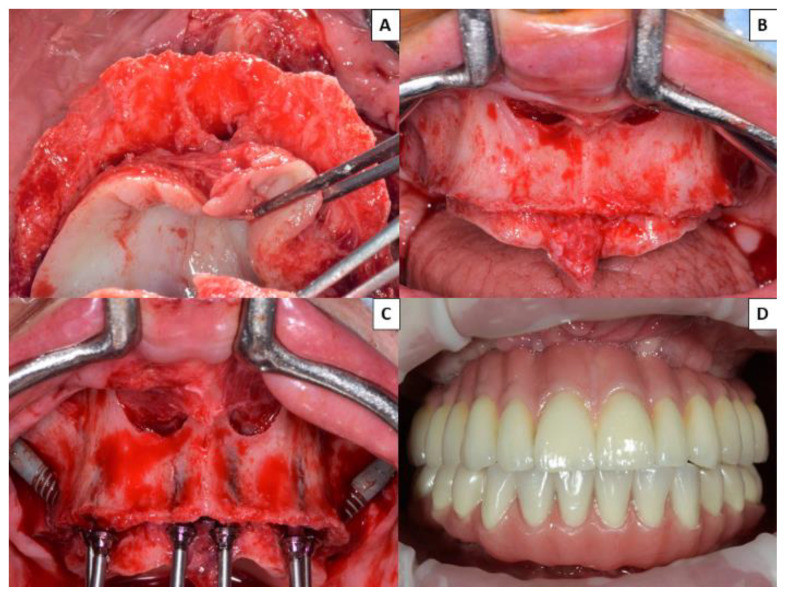
Group 1 patients with atrophic maxilla rehabilitated with two zygomatic implants and four conventional implants in the premaxilla. (**A**) Occlusal image of severe atrophied maxilla. (**B**) Front view of atrophic maxilla. (**C**) Placement of two zygomatic implants and four conventional implants in the anterior region. (**D**) Fabrication of definitive fixed prosthesis 6 months after surgery.

**Figure 3:**
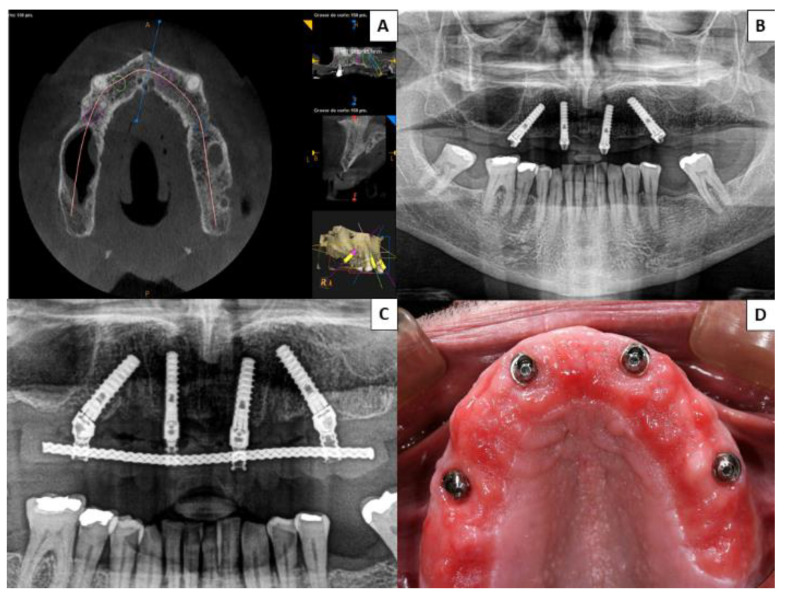
Group 2 patient rehabilitated with four conventional implants in the premaxilla following the all-on-four concept; two placed axially and the other two angled between 35° and 45°. (**A**) Presurgical planning with cone-beam computed tomography (CBCT) of implant distribution. (**B**,**C**) Postsurgical orthopantomographs taken 6 months after surgery to ensure correct fit of definitive prosthesis. (**D**) At a follow-up check-up, a patient complained of food debris trapped beneath the fixed prosthesis; when the prosthesis was unscrewed the patient was found to present mucositis.

No prosthetic complications were recorded in Group 1 patients rehabilitated with zygomatic implants (either in provisional or definitive prostheses) (Figure 2), while three of the 40 patients (7.50%) treated by means of the all-on-four concept suffered prosthetic fractures, and another five (15.50%) presented excessive accumulations of food trapped apical to the definitive prostheses (Figure 3). None of the implants of this study failed, resulting in an implant cumulative survival rate of 100%. The cumulative survival rate for prostheses was 96.25%.

A worse quality of life was shown in the group of patients rehabilitated by means of the all-on-four method in all seven domains of the OHIP-14 and in the overall scores, with statistically significant differences between the two groups (*p* ≤ 0.05) (Table 4).

Patients with zygomatic implants were more satisfied with their prostheses than patients rehabilitated using the all-on-four concept, with statistically significant differences (*p* < 0.001). In fact, 31 out of 40 Group 1 patients (77.50%) stated that they were extremely satisfied with the treatment outcomes (Table 5).

## 4. Discussion

This prospective randomized clinical trial evaluated patients’ quality of life and satisfaction with treatment after rehabilitation of severe atrophic maxillae with fixed prostheses supported by 2–4 zygomatic implants and 2–4 conventional implants (Group 1) compared with four conventional implants in the anterior region following the all-on-four concept (Group 2). Patients were monitored for a minimum of one year after the placement of the definitive prostheses.

Previous studies that compared zygomatic and conventional implants have used various follow-up periods. Aparicio et al. [12] obtained a 100% success rate with 47 zygomatic and 129 conventional implants placed consecutively in 25 patients over a period of 2–5 years. Davo et al. [21] obtained a success rate of 100% with zygomatic implants, while three out of 68 conventional implants (in 18 patients) failed during a follow-up period of 6–29 months. Aparicio et al. [14] conducted another study with a longer follow-up of 12–84 months. Eighty patients received 157 zygomatic implants, of which five failed, and 442 conventional implants of which 20 failed. In general, the technique of zygomatic implants combined with conventional implants in the anterior region is considered a highly predictable treatment obtaining good clinical success rates for rehabilitating edentulous patients with severe maxillary atrophy.

Regarding the all-on-four technique, Patzelt et al. [29] conducted a systematic review of 487 potentially published articles in the MEDLINE database. The 487 articles assessed a total of 4804 implants, of which 2000 were placed in the maxilla. Follow-up times ranged from 12 to 36 months. The review reported a low average failure rate (1.5%). Fifty per cent of these failures occurred in angled implants, while the other 50% were seen in axial implants placed in the anterior region. Most failures took place during the first 12 months. Only 31% of the articles analyzed in the review included follow-up periods of over 36 months.

In the present trial, according to ZAGA classification [13] of the 139 zygomatic implants placed, none of them were classified as ZAGA 0, 3, or 4, while 24 (17.27%) were ZAGA 1 and 115 (82.73%) were ZAGA 2. In the study by Aparicio et al. [17], 200 patients received a total of 198 zygomatic implants, of which 15% were ZAGA 0, 49% ZAGA 1, 20.5% ZAGA 2, 39% ZAGA 3, and 6.5% ZAGA 4.

No prosthetic complications occurred among Group 1 patients rehabilitated with zygomatic and conventional implants (whether provisional or definitive prostheses), while three of the 40 Group 2 patients (7.50%) treated with the all-on-four system suffered fractures of the provisional prostheses and another five (15.50%) presented excessive accumulations of food trapped beneath the definitive prostheses. In most published research, prosthetic fractures in all-on-four systems occur in provisional prostheses without a metallic core. Francetti et al. [25], in a study of 14 patients and 64 implants, documented the fracture of seven acrylic prostheses within four to six months of functional loading. Crespi et al. [26] reported two fractures of acrylic prostheses in a study of 24 patients who received 96 implants, while Hinze et al. [27] reported four fractures of provisional prostheses and one fracture of a definitive metal–resin prosthesis in a study of 19 patients with 76 implants over a 12-month follow-up. Malo et al. [28] recorded eight provisional prosthetic fractures in 18 patients with 72 implants over a 12-month follow-up. In a study that compared immediate loading in 35 patients with immediate provisional prostheses on zygomatic implants with the same loading protocol in 36 patients with six conventional implants, Esposito et al. [15] reported a higher number of prosthetic fractures in patients rehabilitated with conventional implants (*n* = 6) than patients with zygomatic implants (*n* = 1), with a statistically significant difference (*p* = 0.040). However, over a much longer follow-up, Maló et al. [16], in a study of 352 patients with 747 zygomatic implants, described mechanical complications in 156 patients (44%), of which 101 suffered prosthetic fractures in follow-ups of 6 months to 7 years.

In the present trial, 17 of the 139 zygomatic implants (12.23%) showed signs of peri-implantitis, while 19 of the 160 implants placed in the all-on-four system (11.87%) presented peri-implantitis, without significant differences between the two groups (*p* = 0.113). Araújo Nobre et al. [17] recorded a higher percentage of bleeding on probing around zygomatic implants compared with conventional implants, in a clinical peri-implant assessment of the two types of implant. Some authors claim that all-on-four treatments improve plaque and bleeding indices, as the implants are at greater distances from one another, facilitating hygiene maintenance [26]. The main factor conditioning zygomatic implants is whether placement follows an intra-sinus or extra-sinus path. If the implant only has zygomatic anchorage, and the rest of the implant is not surrounded by maxillary bone tissue, its vestibular part will be covered by soft tissue only. This will make it vulnerable to peri-implantitis.

In the present trial, most of the zygomatic implants were classified as ZAGA 2 (82.73%). This could explain the low rate of peri-implantitis. In this sense, studies such as that of Al-Nawas et al. [18] reported high probing depths (6–7 mm) around the machined surfaces of extra-sinus zygomatic implants. The presence of bleeding around extra-sinus zygomatic implants has also been observed by authors such as Maló et al. [19], who reported a high incidence of bleeding on probing the index with these implants over follow-ups of between two months and three years.

No post-surgical complications were observed in the present trial for patients treated with the all-on-four concept, while only two patients treated with zygomatic implants presented complications: 1 (2.50%) case of maxillary sinusitis and another (2.50%) of orbital cellulitis. Sinusitis is considered the most common post-surgical complication affecting zygomatic implants, which appears in between 1.5 and 14% of cases (9–11). It depends largely on the zygomatic implant placement technique used, as extra-sinus implants show a low rate of sinusitis. Studies such as Davó et al. [20] obtained double the incidence of sinusitis as they employed the classic intra-sinus technique. In a 7-year prospective study by Maló et al. [16] with 747 implants, the incidence of maxillary sinusitis was 7%.

In the present trial, Group 2 patients rehabilitated by means of the all-on-four concept reported a worse quality of in all seven domains of the OHIP-14 and in overall scores, with statistically significant difference in comparison with Group 1 rehabilitated with zygomatic implants (*p* ≤ 0.05). However, both groups were shown to have a good oral quality of life (with total scores in OHIP-14 not higher than 31 in any of the study groups), since both treatments (using implant-supported prostheses) are predictable treatment options for improving patient quality of life when compared with conventional complete dentures [30]. In this sense, Dellepiane et al. [35] assessed oral quality of life of patients before, during, and after completion of implant-supported full-arch immediate-loading rehabilitation, using new questionnaires inspired to the OHIP-14. Patients reported an improvement in oral quality of life after full-arch immediate-loading rehabilitation. A statistically significant improvement in aesthetic and chewing ability was found. After four months 92% of the patients did not feel tense with their smile, 96% did not show problems relating with other people or smiling, and 92% did not show difficulty eating certain foods. In addition, patients with zygomatic implants were more satisfied with their prostheses than patients rehabilitated using all-on-four, also with statistically significant difference (*p* < 0.001). Indeed, 31 out of the 40 patients (77.50%) stated that they were extremely satisfied. Further, we must also highlight that the three cases that suffered prosthetic fractures were among the four patients with the worst satisfaction (all-on-four group).

Nevertheless, the trial suffered several limitations. Firstly, the results cannot be compared with any other research because this is the first investigation that compared the quality of life and satisfaction of patients with fixed prostheses supported by zygomatic implants and patients treated with the all-on-four concept. Another important limitation in this study is that both groups presented several differences, including implant type and number of implants; these differences might have affected the outcomes.

The differences between the groups could be due to the fact that with fixed prostheses on zygomatic implants and conventional implants in the premaxilla, less food debris accumulated beneath the prostheses and there were fewer prosthetic fractures. This provided greater confidence in the prostheses, as well as greater comfort. These aspects together led to better emotional and psychological conditions and improved self-esteem. Nevertheless, further long-term clinical studies with larger sample sizes are needed to evaluate the quality of life and satisfaction of patients with fixed prostheses supported by zygomatic implants compared with the all-on-four concept.

## 5. Conclusions

According to the results of this study, rehabilitation of patients with edentulous atrophic maxillae with fixed prostheses supported by zygomatic implants combined with anterior implants in the premaxilla resulted in patients reporting a better quality of life and greater satisfaction with treatment than patients treated with fixed prostheses supported by four anterior implants following the all-on-four concept.

## Figures and Tables

**Table 1:** Study population characteristics.

Patient Sample Characteristics	Group with Zygomatic Implants (*n* = 40)	Group with All-on-Four (*n* = 40)
Follow-up (months): mean ± SD * (range)	19.40 ± 4.37 (12.00–22.00)	20.25 ± 3.01 (12.00–24.00)
Age: mean ± SD	60.18 ± 8.75	60.90 ± 7.01
Sex: *n* (%)		
Male	17 (42.40)	24 (60.00)
Female	23 (57.50)	16 (40.00)
Smoking Status: *n* (%)		
Non-smoker	31 (77.50)	31 (77.50)
≤10	1 (2.50)	3 (7.50)
11–20	5 (12.50)	5 (12.50)
>20	3 (7.50)	2 (5.00)
Alcohol Consumption: *n* (%)		
None	39 (97.50)	35 (87.50)
Daily	0 (0)	0 (0)
Weekend drinker	1 (2.50)	5 (12.50)
Diseases: *n* (%)		
Myocardial infarction	3 (7.50)	4 (10.00)
Hypercholesterolemia	3 (7.50)	4 (10.00)
Arterial hypertension	6 (15.00)	8 (20.00)
Hepatitis B	1 (2.50)	0 (0)
Hepatitis C	2 (5.00)	0 (0)
HIV	1 (2.50)	0 (0)
Epilepsy	1 (2.50)	0 (0)
Cardiac arrhythmias	2 (5.00)	0 (0)
Hypothyroidism	0 (0)	2 (5.00)
Hyperuricemia	1 (2.50)	0 (0)
Occlusion		
Natural teeth	28 (70.00)	24 (60.00)
Metal–porcelain fixed crowns on teeth	1 (2.50)	2 (5.00)
Metal–porcelain fixed crowns on dental implants	9 (22.50)	9 (22.50)
Resin prostheses without dental implants	0 (0)	1 (2.50)
Resin prostheses with dental implants	2 (5.00)	4 (10.00)

* SD = standard deviation.

**Table 2:** Implant distribution.

Characteristics	Total, *n* (%)	Group with Zygomatic Implants, *n* (%)	Group with All-on-Four, *n* (%)
Number of dental implants	396 (100)	236 (100)	160 (100)
Site			
1.1	36 (9.09)	36 (15.25)	0 (0)
1.2	42 (10.61)	2 (0.84)	40 (25.00)
1.3	39 (9.84)	39 (16.52)	0 (0)
1.4	1 (0.25)	1 (0.42)	0 (0)
1.5	77 (19.44)	37 (15.67)	40 (25.00)
1.6	3 (0.75)	3 (1.27)	0 (0)
1.7	1 (0.25)	1 (0.42)	0 (0)
2.1	36 (9.09)	36 (15.25)	0 (0)
2.2	42 (10.61)	2 (0.84)	40 (25.00)
2.3	39 (9.84)	39 (16.52)	0 (0)
2.4	1 (0.25)	1 (0.42)	0 (0)
2.5	77 (19.44)	37 (15.67)	40 (25.00)
2.6	1 (0.25)	1 (0.25)	0 (0)
2.7			
Length	1 (0.25)	1 (0.25)	0 (0)
10 mm	64 (16.16)	20 (8.47)	44 (27.50)
12 mm	154 (38.88)	46 (19.49)	108 (67.50)
14 mm	39 (9.84)	31 (13.13)	8 (5.00)
35 mm	13 (3.28)	13 (5.51)	0 (0)
37.5 mm	6 (1.51)	6 (2.54)	0 (0)
40 mm	40 (10.11)	40 (16.94)	0 (0)
42.5 mm	25 (6.31)	25 (10.59)	0 (0)
45 mm	28 (7.07)	28 (11.86)	0 (0)
47.5 mm	8 (2.02)	8 (3.38)	0 (0)
50 mm	11 (2.78)	11 (4.66)	0 (0)
52.5 mm	2 (0.50)	2 (0.84)	0 (0)
55 mm	3 (0.75)	3 (1.27)	0 (0)
60 mm	3 (0.75)	3 (1.27)	0 (0)
Diameter			
3.5 mm^2^	106 (26.76)	18 (7.64)	88 (55.00)
4.0 mm^2^	151 (38.13)	79 (33.47)	72 (45.00)
4.2 mm^2^	139 (35.11)	139 (58.89)	0 (0)

**Table 3:** Characteristics of dental implants presenting peri-implantitis (Pearson’s χ^2^ test).

Characteristics	Zygomatic Implants (*n* = 139 Implants), *n* (%)	Conventional Implants for All-on-Four (*n* = 160 Implants), *n* (%)
Number of peri-implantitis (*p*-value = 0.113)	17 (100)	19 (100)
Percussion-induced pain (*p*-value = 0.847)		
Yes	4 (23.52)	5 (26.31)
No	13 (76.48)	14 (73.69)
Mobility (*p*-value = 0.615)		
Yes	1 (5.88)	2 (10.52)
No	16 (94.12)	17 (89.48)
Bleeding (*p*-value = 0.087)		
Yes	0 (0)	3 (15.78)
No	17 (100)	16 (84.22)
Suppuration		
Yes	0 (0)	0 (0)
No	0 (0)	0 (0)
Hyperplasia or granuloma		
Yes	0 (0)	0 (0)
No	0 (0)	0 (0)

**Table 4:** Comparison of quality of life between study groups (Student’s *t*-test).

OHIP-14	Group with Zygomatic Implants (*n* = 40) mean ± SD *	Group with All-on-Four (*n* = 40) mean ± SD	*p*-Value
Functional limitation	3.03 ± 1.29	6.35 ± 1.84	<0.001
Physical pain	2.15 ± 0.89	3.80 ± 0.72	<0.001
Psychological discomfort	2.20 ± 1.15	4.23 ± 1.18	<0.001
Physical disability	3.23 ± 1.23	5.08 ± 1.65	<0.001
Psychological disability	3.38 ± 0.71	4.10 ± 1.66	0.013
Social disability	2.60 ± 0.81	4.18 ± 1.31	<0.001
Handicap	1.93 ± 0.69	2.75 ± 0.84	<0.001
Total scores	18.48 ± 3.42	30.43 ± 4.37	<0.001

* SD = standard deviation.

**Table 5:** Patients’ perception of their implant-supported prostheses (Pearson’s χ^2^ test).

Satisfaction (*p*-Value < 0.001)	Group with Zygomatic Implants (*n* = 40), *n* (%)	Group with All-on-Four (*n* = 40), *n* (%)
Extremely satisfied	31 (77.50)	0 (0)
Satisfied	9 (22.50)	6 (15.00)
A little satisfied	0 (0)	30 (75.00)
No change	0 (0)	3 (7.50)
Dissatisfied	0 (0)	1 (2.50)

## Data Availability

The data that support the findings of this study are available from the corresponding author, upon reasonable request.
